# Concerning “Blue Glass”

**Published:** 1877-05

**Authors:** 


### Concerning "Blue Glass."

We are asked why we do not discourse of Pleasonton and "blue glass." Why should we? Is it not abundantly considered by the press already? The object of our pages is to treat of subjects that are too generally neglected; to give expression to those great results of discovery and scientific thought which get but a meagre share of attention from the popular press, and we cannot find half room enough to do this work as it should be done. "But, really, what do you think of Pleasonton, and the blue-glass cure?" is now the obtrusive question. Well, we think that the man is a pestilent ignoramus, and his book the ghastliest rubbish that has been printed in a hundred years. He may be entirely honest, but that is no reason why we should give attention to his egregious folly. Pleasonton, however, it must be confessed, serves one important function: he gauges for us the depth and density of American stupidity. Dr. Morgan says, somewhere, that certain men appear occasionally to play the part of "foolometers" in the community, that is, to measure the number and quality of the fools furnished by any given state of society. Pleasonton has done this for us with an accuracy that leaves nothing to be desired. Our showing in this respect is on a very handsome scale, fully commensurate with the length of the Mississippi, the sweep of the prairies, the glory of the Centennial Exhibition, the grandeur of the national debt, and the splendid proportions of our system of education. He is a public benefactor, in that he has given us another "big thing." The interesting point just now about "blue glass" is psychological. It is an exponent of popular intelligence, an index of culture, a register of common-school work, and a test of the influence of colleges. Our collective schools produce in the community a certain state of mind; "blue glass" indicates it. There is evidently a very close connection here, and the problem deserves to be worked out. If the Intercollegiate Literary Association will offer an additional prize for the best essay on the connection between the study of Latin and Greek and the "blue-glass" mania, THE POPULAR SCIENCE MONTHLY will furnish the money for the purpose.--Prof. YOUMANS, *in Popular Science Monthly for May.*

